# Feedforward regulation of mRNA stability by prolonged extracellular signal-regulated kinase activity

**DOI:** 10.1111/febs.13172

**Published:** 2015-01-08

**Authors:** Takeshi Nagashima, Norihiko Inoue, Noriko Yumoto, Yuko Saeki, Shigeyuki Magi, Natalia Volinsky, Alexander Sorkin, Boris N Kholodenko, Mariko Okada-Hatakeyama

**Affiliations:** 1 Laboratory for Integrated Cellular Systems, RIKEN Center for Integrative Medical Sciences (IMS)Tsurumi-ku, Yokohama, Kanagawa, Japan; 2 Systems Biology Ireland, University College DublinIreland; 3 Department of Cell Biology, University of Pittsburgh School of MedicineS362 Biomedical Science Tower, PA, USA; 4 Conway Institute of Biomolecular & Biomedical Research, University College DublinIreland; 5 School of Medicine and Medical Sciences, University College DublinIreland

**Keywords:** EGFR, ERK, mRNA stability, signal transduction, transcriptome

## Abstract

Extracellular signal-regulated kinase (ERK) plays a central role in signal transduction networks and cell fate decisions. Sustained ERK activation induces cell differentiation, whereas transient ERK results in the proliferation of several types of cells. Sustained ERK activity stabilizes the proteins of early-response gene products. However, the effect of ERK activity duration on mRNA stability is unknown. We analyzed the quantitative relationship between the duration of four ERK activity kinetics and the mRNA expression profile in growth factor-treated cells. Time-course transcriptome analysis revealed that the cells with prolonged ERK activity generally showed sustained mRNA expression of late response genes but not early or mid genes. Selected late response genes decayed more rapidly in the presence of a specific ERK inhibitor than a general transcription inhibitor and the decay rate was not related to the number of AU-rich elements. Our results suggest that sustained ERK activity plays an important role in the lifespan of the mRNA encoded by late response genes, in addition to the previously demonstrated role in protein stabilization of early-response genes, including transcription factors regulating the transcription of mid and late genes. This double-positive regulation of ligand-induced genes, also termed feedforward regulation, is critical in cell fate decisions.

## Introduction

Extracellular signal-regulated kinase (ERK) plays a central role in the signal transduction networks regulating fate decisions in a variety of mammalian cells [1–3]. Ligand stimulation of the membrane receptor tyrosine kinase leads to ERK phosphorylation and activation in the cytosol and then the phosphorylated ERK translocates into the nucleus where it activates the trans-criptional machinery for cell growth, proliferation or differentiation. Interestingly, differences in the duration of ERK activity are often associated with distinct cellular phenotypes. In rat adrenal pheochromocytoma PC12 cells, nerve growth factor (NGF) induces sustained ERK activity for cellular differentiation, whereas epidermal growth factor (EGF)-induced transient ERK activity elicits cell proliferation [4,5]. Similarly, in human breast cancer MCF-7 cells, stimulation with an ErbB3/4 receptor ligand, heregulin (HRG), induces prolonged ERK activity and cellular differentiation, whereas EGF induces transient ERK activity followed by cell proliferation [6,7]. These experimental cell models can prove useful for a quantitative understanding of biological network responses and how this affects cell fate decisions. Previous studies using MCF-7 cells showed that sustained ERK activation induced higher levels of mRNA expression of immediate early genes (IEGs) than short-lived ERK activity [6]. Prolonged ERK activity also stabilized IEG encoded protein products, including the c-Fos transcription factor with a DEF (i.e. a docking site for ERK and FXFP) domain [8,9]. In this network, only sustained ERK activity provides a feedforward AND-gate loop to stabilize the proteins [7], whereas short-lived ERK activity cannot form this loop. Thus, cells with different ERK signal durations induce different protein networks. However, it is unknown whether the sustained ERK activity has any effect on mRNA stabilization itself.

ERK signal duration is determined by several molecular mechanisms. One such example is the positive and negative feedback regulation from ERK to Raf in the Ras–ERK cascade, which induces sustained and transient ERK activity, respectively [5]. Downregulation of the membrane receptors can also contribute to reduction of ERK signal duration. For example, although ligand-receptor binding constants are almost identical for EGF-EGF receptor (EGFR) and HRG-ErbB3/4 receptor binding [10,11], downregulation and deactivation of EGFR activity is much faster than that of the HRG-activated receptors. Rapid downregulation of EGFR activity is caused by endocytosis and degradation of the receptor [12]. The introduction of mutations in the multiple lysine residues within the kinase domain of the EGFR, which are responsible for ubiquitin conjugation, induced sustained activation of EGFR and ERK in response to EGF [13]. Thus, it was demonstrated that the ubiquitination-dependent downregulation of the EGFR is the major mechanism for EGF-induced transient ERK activation. It was also suggested that a mutant ubiquitination-deficient EGFR can be used to modify and sustain the EGF-dependent ERK activation kinetics and therefore to evaluate the effect of ERK signal duration on post-transcriptional regulation of mRNAs.

**Figure 1:**
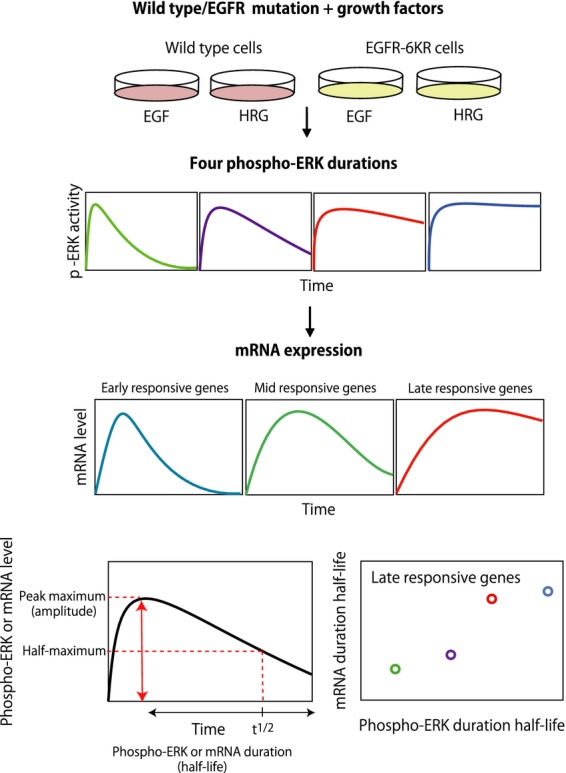
Flow scheme of the study. Growth factor-stimulated wild-type and the EGFR-6KR (ubiquitination deficient-EGFR)-expressing MCF-7 cells were assessed by quantitative time-course analysis of ERK activity and mRNA expression. The relationship between amplitude and duration half-life of the ERK activity and mRNA expression of early, mid, late response genes was analyzed.

In the present study, we generated MCF-7 cells that stably express the 6KR-EGFR mutant, in which the six lysine ubiquitin binding residues are mutated, resulting in sustained activation of ERK in response to EGF. The EGF-evoked ERK profiles, together with the HRG-stimulated ERK profiles in the 6KR and control MCF-7 cells, provided a collection of four ERK temporal profiles that all have the same amplitude but a slightly different duration (Fig.1). The time-course analysis of genome-wide gene expression up to 8 h after ligand stimulation revealed that the cells with prolonged ERK activity showed sustained mRNA expression of the late response genes but not early- to mid-response genes. At 4 h after ligand-stimulation, mRNAs encoded by five out of 10 late response genes decayed more rapidly in the presence of a specific ERK inhibitor than after actinomycin D (ActD) inhibition of total mRNA synthesis, suggesting that prolonged ERK activity might be responsible for mRNA stability. mRNA stability is controlled by gene structure and sequence [14,15] and also by a binding of mRNA stabilizing and destabilizing proteins regulated by ERK [16]. The present study suggests that the cell determination process is a collective multilayered network involving IEG protein stabilization together with stabilization of the late response genes mRNA, and that ERK duration plays a significant role in the process.

## Results

### Ubiquitination-impaired EGFR induces sustained ERK activity

EGF and HRG, growth factor ligands for the EGF and ErbB3/4 receptors, induce transient and sustained ERK activity associated with cellular proliferation and differentiation associated with lipid accumulation of MCF-7 cells, respectively [6]. To systematically analyze the effect of ERK signal duration on mRNA expression dynamics and its relationship with cell fate determination, we first aimed to modify the EGF-triggered ERK signal duration by changing EGFR activation dynamics via impairment of ubiquitination and hence the receptor degradation process. The EGFR possesses a phosphorylation site at tyrosine 1045 that serves as a binding site for the Cbl ubiquitin ligase. However, mutation of this tyrosine is not sufficient to prevent EGFR internalization and degradation [17,18]. On the other hand, mutation of the six lysine residues (6KR; K692, K713, K730, K843, K905 and K946) of the EGFR responsible for ubiquitin conjugation was shown to result in impaired degradation and sustained phosphorylation of the receptor [13,19]. Therefore, we constructed MCF-7 cell lines that stably express 6KR EGFR (6KR) and analyzed their signaling and mRNA expression dynamics in response to EGF and HRG (Fig.1).

**Figure 2:**
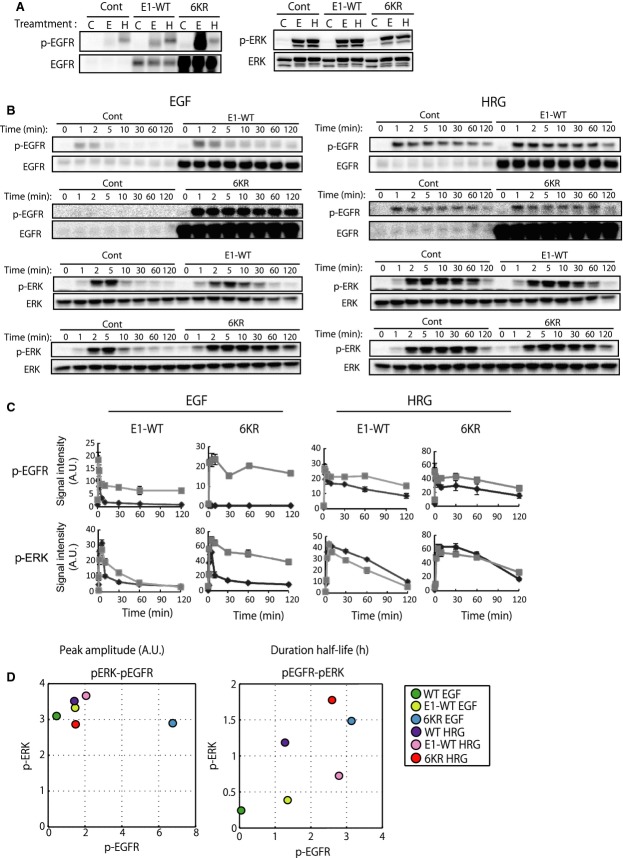
Phosphorylation of EGFR and ERK in control, wild-type EGFR (E1-WT) or 6KR-EGFR (6KR) expressing MCF-7 cells in response to EGF and HRG stimulation. (A) Levels of EGFR phosphorylation (at 2 min) (left) and ERK phosphorylation (at 5 min) (right) in the three cell lines. C, control, no stimuli; E, EGF; H, HRG). (B) Time-course phosphorylation of EGFR and ERK for up to 2 h of ligand EGF (left) and HRG (right)-stimulated cells. Time-course cell lysates of control MCF-7 cells and E1-WT or 6KR cells were loaded side by side. Representative images for two independent experiments are shown. (C) Time-course graphic representation of the data in (B). Black lines, control cells; grey lines, E1-WT or 6KR cells. (D) Quantitative relationship between EGFR and ERK phosphorylation. Amplitudes of EGFR and ERK phosphorylation obtained from (A) were plotted (left). Duration half-lives of phosphorylation were calculated from the phosphorylation time-course (B), as described in the Materials and methods, and then plotted (right). Colored circles show the corresponding cells and conditions. Average values of two independent experiments are shown.

When compared with control MCF-7 cells (expressing empty vector) and the cells expressing wild-type EGFR (E1-WT), EGFR phosphorylation in 6KR cells was markedly elevated (Fig.2A), and the time-course pattern in response to EGF was sustained (Fig.2B,C). On the other hand, although E1-WT showed a higher amplitude of EGFR phosphorylation than the control cells (Fig.2A), the stability of ERK was not altered significantly (Fig.2B). Phosphorylation of ERK in the EGF-stimulated 6KR cells became sustained and was comparable to the HRG response in 6KR and control cells (Fig.2B). Interestingly, the amplitude of ligand-stimulated ERK phosphorylation was almost the same under all conditions (Fig.2A), although the duration varied (Fig.2B,C). To quantitatively evaluate the signaling properties of the different cells, we calculated the phosphorylation amplitudes of EGFR at 2 min and ERK at 5 min, as well as the duration half-lives of these kinase activities (for details see Materials and methods). We could observe reasonable positive correlations between EGFR and ERK for their phospho-duration half-lives but no such trend for the amplitudes (Fig.2D).

### Genes responsible for cell differentiation are expressed at later time points

**Figure 3:**
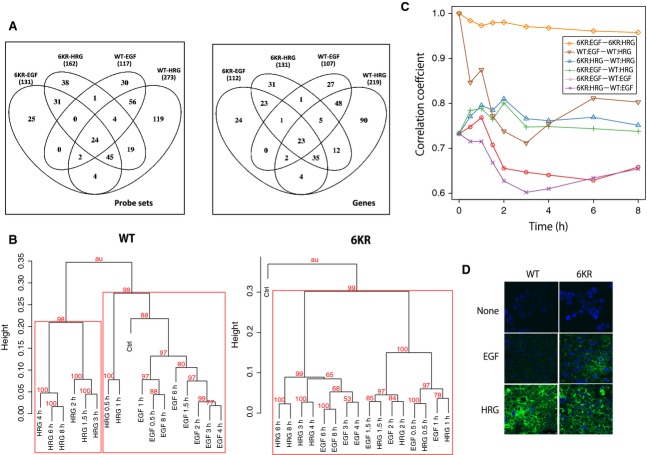
Transcriptome analysis of EGF or HRG-stimulated wild-type and 6KR MCF-7 cells. (A) Genes whose expression levels at the measured time points (0.5, 1, 1.5, 2, 3,4, 6 and 8 h) were significantly changed (FDR < 0.01) from the nonstimulated cells (0 time point) were extracted with rankprod. The sum of genes at all time points is shown. Probe set number (left) and gene number (right). (B) Cluster dendrogram of the above genes (FDR < 0.01) for wild-type (left) and 6KR cells (right). Distance: 1 – correlation coefficient, Clustering method; average linkage, approximate unbiased (AU) values (%) are shown. A larger AU value indicates stronger support of the cluster by data. (C) Time-course change in Pearson's correlation coefficient for each cell and condition. (D) Cell differentiation after growth factor-stimulation of wild-type and 6KR MCF-7 cells analyzed by BODIPY staining.

**Figure 4:**
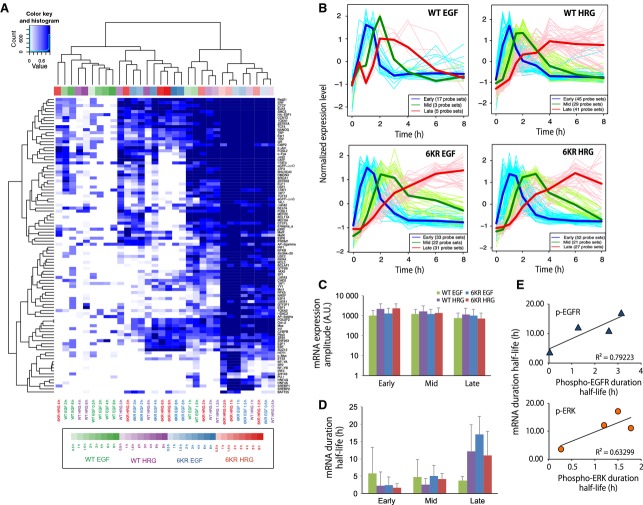
Prediction of transcription factor binding sites and mRNA expression dynamics. (A) TFBS in growth factor-regulated genes (FDR < 0.01) were predicted, as described in the Materials and methods, and shown in a heatmap. Each cell type and condition is colored differently. WT-EGF, green; WT-HRG, purple; 6KR EGF, blue; HRG 6KR, red. The darkness of the color bars corresponds to the time after growth factor stimulation. Cells in the heatmap are color coded according to the enrichment score, which is defined as 1 – FDR, with a darker color indicating higher enrichment. (B) Time-course patterns of early (blue), mid (green) and late (red) upregulated gene groups in WT-EGF, WT-HRG, 6KR EGF and 6KR HRG cells. The thin lines represent the expression patterns of the individual probe set, whereas the thick line represents their average. (C) Average mRNA expression amplitude of early, mid and late genes in WT-EGF (green), WT-HRG (purple), 6KR EGF (blue) and 6KR HRG (red). (D) Average mRNA expression half-life (h) of early, mid and late genes for each cell type and condition. Error bars denote the SD of the gene numbers shown in (B). (E) Graphic representation of the relationship between EGFR (top) and ERK (bottom) duration half-lives of phosphorylation (*x*-axis) and late gene mRNA duration half-lives (*y*-axis). Only average values are shown in the graph. Exponential curves were fitted to the data.

To identify genes for which expression was signal-responsive, we next performed time-course gene expression analysis for up to 8 h on the EGF- and HRG-treated 6KR cells using Affymetrix microarrays and compared this with wild-type MCF-7 cells. We confirmed that the wild-type cells and control cells expressing empty vector showed identical kinetics of signaling and representative gene expression [6]. We found significant expression changes [false discovery rate (FDR) < 0.01] in 107 (117 probe sets) and 219 (273 probe sets) genes in the wild-type cells and 112 (131 probe sets) and 131 (162 probe sets) genes in the 6KR cells after EGF- or HRG-treatment, respectively (Fig.3A). Cluster analysis of these genes showed that, in wild-type cells, the EGF and HRG response gene profiles become significantly different after approximately 1.5 h, whereas, in the 6KR cells, their responses remained in a single cluster throughout this period (Fig.3B). The time-course trajectory of Pearson's correlation coefficients confirmed a moderate correlation between 6KR and wild-type cells at basal level (∼ 0.73), a high correlation coefficient (> 0.9) between EGF- and HRG-6KR cells (Fig.3C), and a constant correlation between 6KR-EGF and WT-HRG or 6KR-HRG and WT-HRG cells. Lipid staining showed that, unlike the wild-type cells, the EGF-treated 6KR cells acquired the ability to accumulate lipid droplets, a sign of MCF-7 cell differentiation, as seen in the HRG-treated wild-type and 6KR cells (Fig.3D). These analyses showed that the EGF-stimulated 6KR cells are similar to the HRG-stimulated wild-type cells in terms of ERK signal duration, gene expression signatures and cellular phenotype. The results also indicated that the dynamics of those cellular events might be strongly related, implying that the duration of ERK signaling activity, and not the ligand type, is a strong determinant of gene expression and cell fate decisions. We examined the upstream transcription factor binding sites (TFBS) located up to 2 kbp upstream of transcription start sites predicted from public databases for the above genes (see Materials and methods). This analysis suggested that genes regulated at the different time points are marked by a variable number of regulatory sites, although the four cell conditions share many common transcription factors at the early time points (light colors, each color represents each cell and condition) but fewer later on (dark colors) (Fig.4A). Particularly, in WT-EGF cells, a condition under which cell differentiation is not observed, the mid to late genes showed TFBS combinations distinct from other cell conditions, whereas the early TFBS were rather common. This analysis suggested that the genes responsible for differentiation might be expressed at later rather than early time points.

### ERK duration and late mRNA duration are correlated

We next attempted to uncover a quantitative relationship between signaling dynamics and upregulated gene expression dynamics. We conventionally classified the genes into early (expression peak appears within ∼ 1.5 h), mid (∼ 2–4 h) and late (> 4 h) groups using a clustering method (Fig.4B and Table S1). The mean expression mRNA amplitudes were in a similar range under all cell conditions (Fig.4C). On the other hand, the mean mRNA duration half-lives of early- and mid-response genes were relatively similar for all conditions, whereas mRNA duration half-lives of the late genes varied and showed a moderate correlation with the duration half-lives of ERK and EGFR phosphoproteins (Fig.4D,E).

**Figure 5:**
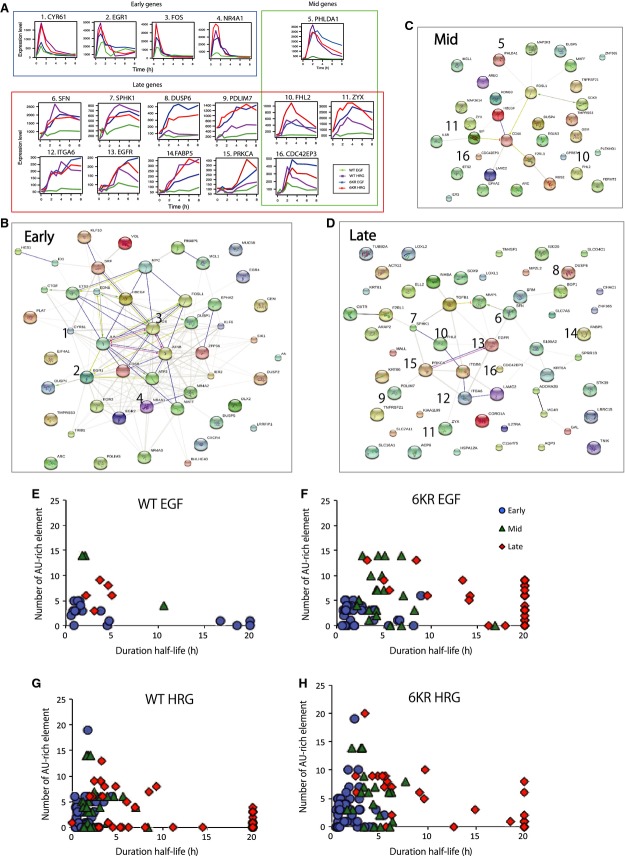
Network and functional analysis of early, mid and late genes. (A) Representative mRNA expression patterns of early, mid and late genes. (B–D) Early, mid and late group genes in each cell condition were added together for each time group and analyzed using the STRING database for molecular interactions (for annotation of each symbol, see: http://string-db.org/). The numbered genes show mRNA expression profiles of the time-course microarray in (A); WT-EGF (green), WT-HRG (purple), 6KR EGF (blue) and 6KR HRG (red). *CDC42EP3*,*FHL2* and *ZYX* were selected as mid or late response genes in a condition-dependent manner. (E–H) Relationship between the number of ARE motifs (ATTTA) and mRNA expression duration half-life in WT-EGF (E), WT-HRG (F), 6KR-EGF (G) and 6KR-HRG (H) cells. Early (blue), mid (green) and late (red) genes are shown.

The mRNA expression profiles clearly showed that early response genes had transient patterns for all cell conditions, whereas the mid-to-late response genes showed more diverse patterns (Fig.5A). Molecular network and gene ontology analysis using the STRING database [20] suggested an interconnected molecular regulatory network of transcription factors *ATF3*,*FOS*,*FOSB*,*JUN* and *JUNB* in the early gene groups (Fig.5B), which is consistent with the molecular function enrichment analysis for RNA polymerase II-dependent transcription (Table1). The early and mid genes were enriched for negative regulators of signal transduction pathways. On the other hand, the late genes group was enriched with genes encoding signaling proteins, as well as positive regulators of cell migration and motility (Fig.5C,D). These late genes formed a network centering on *EGFR* (Fig.5D) and showed enriched function within the MAPK cascade (Table1). In the EGF-WT cells, all of these late genes (five out of five genes) [*CDC42EP3* (Cdc42 effector protein 3), *EGFR*,*F2RL1* (protease-activated receptor 2, PAR-2), *SOX9* (transcription factor, Sox9) and *TNFRSF21* (tumor necrosis factor receptor-related death receptor 6)] had multiple AU-rich element (ARE) motifs in their 3′ UTR (Table S1), the presence of which accelerates mRNA degradation [14,21]. Unexpectedly, the same genes showed relatively sustained mRNA expression patterns under other cell conditions where ERK activity was prolonged (Fig.5A). We analyzed the relationship between the ARE ATTTA motif numbers and the mRNA half-lives of early, mid and the late genes in each cell type and under each condition (Fig.5E–H). The results indicated that early genes generally have a short mRNA half-life, even if they do not have many ARE motifs (Fig.5E–G). Mid and late genes with more ARE motifs had shorter mRNA half-lives (Fig.5E–H). However, the mRNA stability of late genes without ARE still showed considerable variability (Fig.5F). The data suggested that the mRNA duration of ligand response genes is not determined by the presence of ARE.

**Table 1:** Molecular function enrichment analysis of early, mid and late genes. Analysis was performed using the STRING database. Gene ontology biological processes of the top 15 enriched functions are shown (*P*-value, without correction). MAP, mitogen-activated protein; MAPK, mitogen-activated protein kinase

Term	Number of genes	*P*-value (not corrected)
Early responsive genes		
Negative regulation of cellular process	26	4.63 × 10^−11^
Regulation of transcription from RNA polymerase II promoter	18	1.04 × 10^−10^
Negative regulation of biological process	26	3.07 × 10^−10^
Blood vessel development	12	3.45 × 10^−10^
Vasculature development	12	5.96 × 10^−10^
Tissue development	17	9.74 × 10^−10^
Anatomical structure formation involved in morphogenesis	14	1.28 × 10^−9^
Regulation of signaling	21	1.57 × 10^−9^
Transcription from RNA polymerase II promoter	12	1.79 × 10^−9^
Response to organic substance	20	4.72 × 10^−9^
Cardiovascular system development	13	6.15 × 10^−9^
Circulatory system development	13	6.15 × 10^−9^
Negative regulation of macromolecule metabolic process	17	9.27 × 10^−9^
Blood vessel morphogenesis	10	1.08 × 10^−8^
Negative regulation of cellular macromolecule biosynthetic process	14	2.22 × 10^−8^
Mid responsive genes		
Negative regulation of intracellular protein kinase cascade	5	2.30 × 10^−6^
Negative regulation of response to stimulus	9	3.96 × 10^−6^
Negative regulation of signal transduction	8	7.80 × 10^−6^
Negative regulation of MAPK cascade	4	9.98 × 10^−6^
Negative regulation of signaling	8	1.34 × 10^−5^
Negative regulation of cell communication	8	1.42 × 10^−5^
Regulation of MAPK cascade	6	1.97 × 10^−5^
Regulation of intracellular protein kinase cascade	7	5.86 × 10^−5^
Regulation of signaling	12	6.43 × 10^−5^
Regulation of signal transduction	11	8.99 × 10^−5^
Tissue development	9	1.17 × 10^−4^
Regulation of cell communication	10	1.60 × 10^−4^
Endoderm development	3	1.73 × 10^−4^
Negative regulation of MAP kinase activity	3	1.83 × 10^−4^
Regulation of response to stimulus	12	2.69 × 10^−4^
Late responsive genes		
MAPK cascade	7	2.12 × 10^−6^
Protein deamination	2	1.10 × 10^−5^
Negative regulation of immune system process	6	1.19 × 10^−5^
Epidermis development	7	1.22 × 10^−5^
Regulation of anatomical structure morphogenesis	9	2.83 × 10^−5^
Cell surface receptor signaling pathway	17	3.09 × 10^−5^
Positive regulation of norepinephrine secretion	2	3.31 × 10^−5^
Regulation of immune system process	11	3.51 × 10^−5^
Positive regulation of cell migration	6	4.28 × 10^−5^
Cell migration	9	4.39 × 10^−5^
Positive regulation of cell motility	6	4.66 × 10^−5^
Positive regulation of locomotion	6	5.52 × 10^−5^
Positive regulation of multicellular organismal process	8	5.76 × 10^−5^
Regulation of body fluid levels	9	5.82 × 10^−5^
Positive regulation of cellular component movement	6	5.83 × 10^−5^

### Sustained cytosolic ERK activity is responsible for the stability of mRNA encoded by late response genes

Late genes demonstrating long lasting expression patterns might be constantly transcribed in an ERK-dependent manner. Alternatively, ERK might regulate the mRNA degradation process. To clarify what determines the prolonged mRNA presence, we compared the mRNA decay (decay half-life) of these genes under control conditions and after cell perturbations. We applied ActD (a DNA-dependent RNA synthesis inhibitor that blocks activity of RNA polymerase) or U0126 [a mitogen-activated protein kinase kinase (MEK) inhibitor] after 4 h of ligand stimulation. We used 5 μg·mL^−1^ ActD, which is sufficient to inhibit RNA synthesis in mammalian cells [22]. We selected 10 late response genes {[*CDC42EP3*,*DUSP6* (MAP kinase phosphatase 3), *EGFR*,*FABP5* (fatty acid-binding protein 5), *ITGA6* (integrin α-6), *LAMC2* (laminin gamma 2), *PDLIM7* (PDZ and LIM domain 7), *PRKCA* (protein kinase C, α), *SFN* (stratifin, 14-3-3 sigma), *SPHK1* (sphingosine kinase 1), *ZYX* (zyxin)] and a mid-to-late gene, *FHL2* (LIM domain protein DRAL)} in the EGFR interaction network for analysis (Fig.5D). We selected these genes because they showed a significant change in expression levels in response to growth factor stimulation and also had a relatively abundant gene expression level under the basal (without stimuli) condition; thus, we could detect small changes in mRNA levels caused by the small molecule inhibitors.

Overall, mRNA stability of *CDC42EP3* and *DUSP6* was very sensitive to these inhibitors and showed rapid decay (Table2 and Fig. S1). On the other hand, *FABP5*,*FHL2* and *ZYX* were relatively resistant to the inhibitor treatment. However, close examination of mRNA duration showed that there are ligand- and cell-dependent preferences for inhibitor-mediated mRNA decay. For example, *DUSP6* mRNA decayed more rapidly in the presence of U0126 than with ActD treatment in EGF-WT and 6KR cells. *FHL2* mRNA levels were not significantly changed by those inhibitors but showed slightly more rapid decay in the presence of U0126 in EGF-WT and 6KR. *PDLIM7* and *SFN* mRNA was more sensitive to U0126 in 6KR but showed no significant differences in wild-type cells. On the other hand, *EGFR* and *PRKCA* mRNA rapidly decayed in all ActD-treated cells.

**Table 2:** qRT-PCR analysis to determine the mRNA decay half-life of representative late response genes. The cells were stimulated with growth factor ligands (EGF or HRG) for 4 h, and then ActD (mRNA synthesis inhibitor) or U0126 (MEK inhibitor). The mRNA decay rate (decay half-life) was derived as described in the Materials and methods. The data are the mean ± SD. NA, not applicable. The shortest duration of ligand/inhibitor combination in each cell type is shown in bold

			mRNA decay half-life (h)				
Gene	Cell type	Ligand	Ligand only	Ligand + ActD	Ligand + U0126	mRNA stability	Number of ‘ATTTA’ motif
*CDC42EP3*	WT	EGF	8.58 ± 0.82	**1.22 ± 0.2**	1.62 ± 0.27	ERK and transcription equally-dependent	6
		HRG	3.13 ± 0.05	1.26 ± 0.12	**1.25 ± 0.16**		
	6KR	EGF	4.55 ± 0.4	**1.37 ± 0.07**	1.38 ± 0.06		
		HRG	5.04 ± 0.13	1.72 ± 0.02	**1.55 ± 0.04**		
*DUSP6*	WT	EGF	NA	2.31 ± 0.17	**1.44 ± 0.07**	More ERK-dependent	1
		HRG	4.91 ± 0.91	**1.02 ± 0.05**	1.05 ± 0.21		
	6KR	EGF	2.81 ± 0.14	1.21 ± 0.01	**1.07 ± 0.34**		
		HRG	4.83 ± 0.06	1.73 ± 0.13	**0.9 ± 0.13**		
*EGFR*	WT	EGF	> 20	**4.97 ± 0.06**	16.5 ± 6.09	More transcription-dependent	9
		HRG	16.31 ± 0.35	**6.99 ± 0.08**	7.76 ± 0.1		
	6KR	EGF	> 20	**3.74 ± 0.1**	16.98 ± 1.63		
		HRG	NA	**4.24 ± 0.02**	> 20		
*FABP5*	WT	EGF	NA	> 20	NA	Neither	0
		HRG	NA	NA	NA		
	6KR	EGF	NA	NA	NA		
		HRG	0.69 ± 0	0.69 ± 0	0.69 ± 0		
*FHL2*	WT	EGF	4.37 ± 0.12	**3.17 ± 0.01**	3.5 ± 0.14	More ERK-dependent	6
		HRG	4.3 ± 0.17	2.29 ± 0.08	**2.19 ± 0.02**		
	6KR	EGF	2.13 ± 0.1	1.77 ± 0.06	**1.73 ± 0.03**		
		HRG	2.56 ± 0	2.32 ± 0.1	**1.96 ± 0.05**		
*PDLIM7*	WT	EGF	> 20	6.54 ± 0.34	**6.01 ± 0.69**	More ERK-dependent	0
		HRG	> 20	8.23 ± 0.01	**5.37 ± 0.1**		
	6KR	EGF	> 20	16.11 ± 0.46	**5.01 ± 0.03**		
		HRG	NA	N.A	**15.16 ± 3.55**		
*PRKCA*	WT	EGF	NA	**10.21 ± 2.81**	NA	More transcription-dependent	7
		HRG	NA	**4.06 ± 0.21**	NA		
	6KR	EGF	NA	NA	NA		
		HRG	NA	**> 20**	NA		
*SFN*	WT	EGF	NA	10.16 ± 0.29	**6.31 ± 0.27**	More ERK-dependent	0
		HRG	6.18 ± 0.96	6.18 ± 0.91	**4.63 ± 1.32**		
	6KR	EGF	6.42 ± 0.5	7.55 ± 0.1	**3.36 ± 0.12**		
		HRG	17.37 ± 4.27	> 20	**7.72 ± 1.07**		
*SPHK1*	WT	EGF	> 20	**3.9 ± 0.51**	4.77 ± 0.14	More transcription-dependent	0
		HRG	> 20	**2.76 ± 0.88**	7.18 ± 2.86		
	6KR	EGF	11.63 ± 15.47	**3.47 ± 3.93**	4.48 ± 5.35		
		HRG	6.02 ± 7.54	**3.58 ± 4.09**	6.87 ± 8.74		
*ZYX*	WT	EGF	7.34 ± 1.24	**4.47 ± 0.41**	5.55 ± 1.4	More ERK-dependent	0
		HRG	7.87 ± 2.06	8.29 ± 3.87	**6.6 ± 2.54**		
	6KR	EGF	4.13 ± 0.69	4.7 ± 0.78	**3.09 ± 0.66**		
		HRG	2.57 ± 2.65	3.55 ± 4.04	**2.45 ± 2.49**		

**Figure 6:**
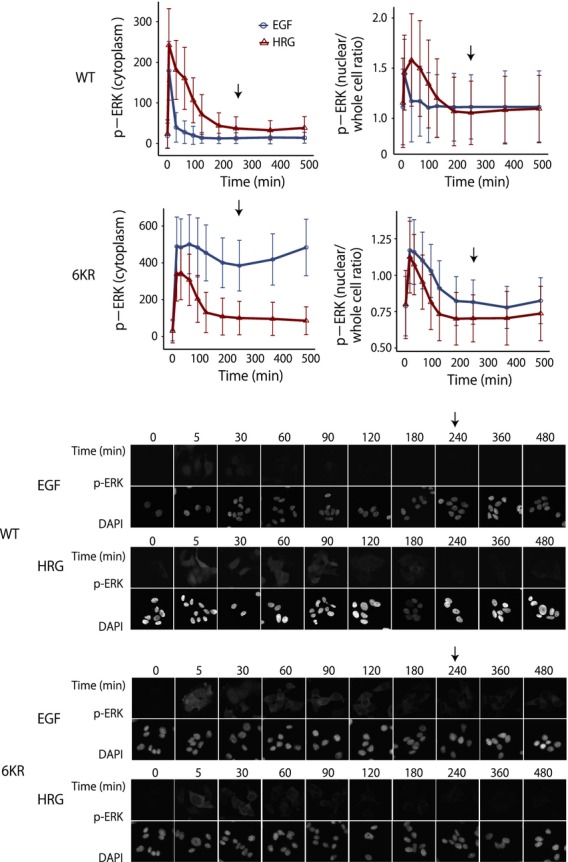
Time-course analysis of phospho-ERK localization at the single cell level. (A) Quantification of phospho-ERK in cytosol (left) and nuclear/whole cell ratio (right) of wild-type (upper) and 6KR (bottom) cells up to 8 h after ligand stimulation. At each time point, 1200–2000 cells were analyzed and average values were obtained. The bar denotes the SD. EGF (blue) and HRG (red). Arrow indicates the 4-h time point. (B) Representative images of phospho-ERK immunostaining (top) and DAPI staining (middle) for each condition.

Although there is a significant gene-to-gene variation, *DUSP6*,*FHL2*,*PDLIM7*,*SFN* and *XYZ* showed more rapid mRNA decay after addition of the MEK inhibitor than after ActD treatment (Table2 and Fig. S1), implying that their mRNA decay rates are more sensitive to ERK-dependent mRNA stabilization than transcription. To confirm that the ERK-dependent prolonged mRNA profile is a result of mRNA stabilization in the cytosol and not ERK-controlled transcription in the nucleus, we carried out immunostaining to quantify the localization of phosphorylated ERK. The cytoplasmic ERK phosphorylation remained relatively high in HRG-treated cells and EGF-stimulated 6KR cells after 4 h (Fig.6A, left), which is consistent with the results of western blot analysis (Fig.2B,C). On the other hand, the phosphorylated nuclear ERK levels returned to the basal level (equivalent to the values at 0 time point) after 4 h of ligand stimulation in the EGF- and HRG-treated wild-type cells and 6KR cells (Fig.6), suggesting that no (or at least minimal) transcription, is regulated by ERK at this time point. These results, together with the mRNA duration profiles of late genes (Fig.5A) and the mRNA decay rate (Table2), suggest that the mRNA stability of late expressing genes is likely controlled by cytosolic ERK activity.

## Discussion

Although ERK is a well-known regulator of gene transcription, it can also control mRNA stability. Transcripts of genes such as *dusp1*/*MKP-1* [23], *dusp6*/*MKP-3* [24], *VEGF* [25], *COX-2* [26], *FAK* [27] and *p21* [28] have shown prolonged mRNA expression in the presence of active ERK in different types of cells [16]. The stabilization of these mRNAs was regulated at the 3′ UTR by the RNA stabilizing protein HUR/ELAVL1 [23,28] and destabilizing proteins tristetraprolin (TTP/ZFP36)[24,25], PUM2 [24] or ARE/poly(U)-binding factor 1 (AUF1) [26] in an ERK-dependent manner. However, those studies did not examine the quantitative relationship between dynamics of ERK activity and its target mRNA expression. The duration of ERK activity is indeed important for mammalian cell fate determination. Numerous studies have demonstrated the molecular mechanisms and regulatory logic that shape distinct ERK dynamics in the signaling network [4,5,7]. However, it remained unclear whether ERK signal duration had any effect on genome-wide mRNA expression dynamics.

In the present study, we focused on ligand-stimulated ERK signaling and subsequent gene expression and assessed the quantitative relationship between ERK activity and its target mRNA expression. Our analysis showed that sustained ERK activity was associated with prolonged mRNA expression of late response genes that peaked after 4 h but not with early or mid response genes. In addition, although whole cell ERK activity was sustained, nuclear ERK activity was transient, disappearing within 4 h. This is considered to be a result of the activity of newly synthesized DUSPs (ERK phosphatases) localized in the nucleus [7,29–31]. Thus, the overall picture indicated that transient nuclear ERK activity contributes to the initiation of mRNA transcription, whereas prolonged cytosolic ERK activity contributes to the mRNA stabilization encoded by late response genes.

Interestingly, mRNA stability and the function of the protein encoded by the mRNA appears to be closely related to cellular function. For example, gene expression dynamics studies using tumor necrosis factor-α-treated 3T3 fibroblasts showed that late genes (peaking at 12 h) containing fewer AREs have a higher mRNA stability than the early (0.5 h) and mid (2 h) genes and that the function of these late response genes is related to inflammation, the typical response to this ligand [32]. In addition, a study using mouse fibroblasts, human B cells and differentiating mouse embryonic stem cells provided further evidence suggesting that short-lived mRNAs are enriched for transcriptional regulation and signal transduction genes, whereas long-lived mRNA is enriched for cellular respiration, energy metabolism, translation, etc. [33,34]. These studies indicate that mRNAs encoding transcriptional regulatory proteins generally have short half-lives, whereas mRNAs related to cellular differentiation tend to have a slow decay. Consistent with these findings, we also found that early to mid response genes in MCF-7 cells with short half-lives are enriched in functions related to transcriptional regulation and negative regulation of signal transduction, whereas delayed expressing genes, for which the mRNAs are supposed to be stabilized, are enriched for the positive regulation of development, cell migration, locomotion, morphogenesis and the positive regulation of signaling pathways (Fig.5 and Table1). Consequently, these late response genes might help promote differentiation of MCF-7 cells.

**Figure 7:**
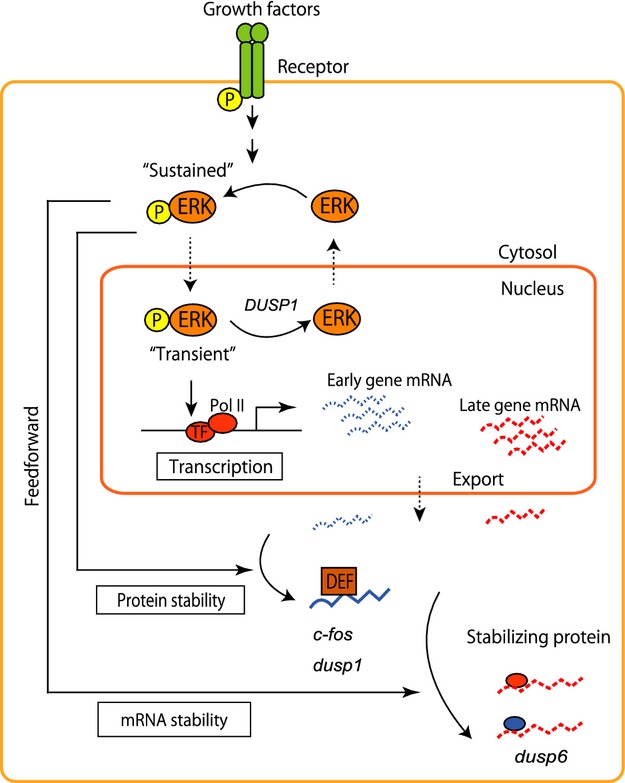
Model for multiple regulation of mRNA and protein stabilities mediated by sustained ERK activity. Membrane receptor-dependent ERK activation induces its translocation to the nucleus and activation of transcription factors for the expression of early, mid and late genes. Early response gene (ERG) products, whose encoding genes have DEF (i.e. a docking site for ERK and FXFP) domain, are stabilized by sustained ERK activity in the cytosol. The present study showed that the prolonged cytosolic ERK activity also stabilizes the late response mRNAs. In both cases, the sustained ERK activity comprises feedforward AND-gate loops, where the duration of ERK activity is critical for late genes and cell fate decisions.

In addition, further evidence suggests that kinase activity plays an important role in prolonged subsequent mRNA expression for cell differentiation. *IL-2* mRNA, a cytokine responsible for cell differentiation in T cell activation, represents one such example [35]. *IL-2* mRNA contains several AREs in the 3′ UTR and has a short half-life in resting T cells; however, *IL-2* mRNA expression is prolonged upon T-cell activation but only when upstream c-Jun N-terminal kinase (JNK) is activated [36]. Togther with the results of the present study, this suggests that, although there are differences in species of kinases in each signaling network, kinases such as ERK and JNK activate corresponding transcription factors and also stabilize newly synthesized mRNAs. Thus, mRNA stability might be determined by the interplay between the kinase and the target mRNAs, where only sustained kinase activity forms a feedforward loop able to stabilize mRNAs and prolong their expression. The findings of the present study and those of earlier studies suggest that sustained cytosolic ERK activity might control both post-translational regulation of IEG products (*c-fos*,*dusp1*, etc.) and post-transcriptional regulation (mRNA stability) of the late expression genes (Fig.7).

Overall, the present study using the ERK signaling system shows that signaling dynamics and mRNA dynamics are closely related, and that signal duration, rather than its amplitude, is important for mRNA stability and cell determination.

## Materials and methods

### Cell culture and treatment

MCF-7 cells were maintained in DMEM medium (Gibco BRL, Gaithersburg, MD, USA) supplemented with 10% FBS. Prior to growth hormone treatment, the cells were serum-starved for 16–24 h, and then EGF (PeproTech House, London, UK) or HRG-β 176-246 was added for further analysis. After incubation with the growth factors for the indicated time, the cells were washed twice with PBS. In the case of inhibitor assays, cells are treated with 500 nm of U0126 (Calbiochem, San Diego, CA, USA) or 5 μg·mL^−1^ of actinomycin D (Wako Pure Chemical Industries, Tokyo, Japan) for the indicated times. Cells were then lysed with Bio-Plex lysis buffer (Bio-Rad Laboratories, Hercules, CA, USA) for western blot analysis. Cells were lysed with RNA lysis buffer (Nucleo Spin RNA II; Macherey-Nagel GmBH & Co., Düren, Germany) for quantitative real time-PCR (qRT-PCR).

### Construction of plasmids and stable cell lines

A human wild-type EGFR expression plasmid (pCMV-E1wt) was constructed. The coding sequence of EGFR (a gift from Dr S. Yokoyama, RIKEN) [37] was amplified by PCR. The PCR product was introduced into an *Eco*RV-*Xba*I restriction site in the pCMV-Neo-6 vector (OriGene, Rockville, MD, USA). A plasmid containing the EGFR-6KR mutant was constructed as described previously [13]. With the plasmid as a template, an EGFR-6KR gene was cloned into pCMV-Neo-6 vector using the same primers indicated in the pCMV-E1wt section. The resulting plasmid was named pCMV-E1-6KR. MCF-7 cell lines over-expressing receptors were established by transfecting with either the plasmids reported above or the pCMV-Neo-6 vector as a control cell line. Stable receptor clones containing those plasmids were selected and maintained by treating with 200 μg·mL^−1^ G418. For selection of the clones, EGFR expression and phosphorylation of EGFR and ERK in response to growth factor stimulation were examined by western blotting.

### Immunoblotting

Cell lysates was cleared by centrifugation and the protein concentration of the supernatant was determined using a DC protein assay reagent (Bio-Rad Laboratories). Levels of phosphorylated and total proteins were analyzed by western blotting. For western blot analysis, anti-phospho-EGFR (pY1068), anti-ERK (p44/42 MAP kinase) and anti-phospho-ERK (Thr202/Tyr204) were purchased from Cell Signaling Technology, Inc. (Beverly, MA, USA). Anti-EGFR antibody was purchased from Fitzgerald Industries (North Acton, MA, USA). The protein band intensities were quantified using a densitometer (Fuji Film Corp., Tokyo, Japan or ImageQuant LAS4000; GE Healthcare, Milwaukee, WI, USA). In all of the reported results, error bars denote the SE for at least two independent experiments.

### Microarray analysis

The gene expression analysis was performed with the wild-type and 6KR MCF-7 cells treated with 10 nm EGF or HRG for 0.5, 1, 1.5, 2, 3, 4, 6 and 8 h. Total RNA was isolated using Trizol reagent (Invitrogen, Carlsbad, CA, USA) and then purified using the RNeasy Mini kit (Qiagen, Valencia, CA, USA). RNA quality was assessed using a Bioanalyzer (Agilent Technologies, Santa Clara, CA, USA). First- and second-strand complementary DNA (cDNA) synthesis, biotin-labeled cRNA synthesis, fragmentation of cRNA and hybridization reactions were performed using a one cycle cDNA synthesis kit (Affymetrix, Santa Clara, CA, USA). GeneChip (Affymetrix U133A 2.0 chip for parental MCF-7 and U133 Plus 2.0 chip for 6KR) experiments were carried out in accordance with the manufacturer's instructions (two chips per every time point). Scanned images were processed by RMA implemented as a justRMA function in the affy package to determine gene expression levels. To compare two different chips, 22 000 probe sets commonly found in both the U133 Plus 2.0 and U133A2 chip were used in the subsequent analyses. Microarray data used in the present study was deposited in GEO (Gene Expression Omnibus) database (GSE13009 for parental MCF-7 cells and GSE57547 for 6KR cells). Genes for which the expression levels were altered relative to the nontreated cells after growth factor treatment were extracted using rankprod (FDR < 0.01). Time- and ligand-dependent expression profiles of differentially expressed genes were analyzed by hierarchical clustering using pvclust (clustering method: average linkage, distance: 1 – correlation coefficient). Gene expression time courses were then analyzed by hierarchical clustering. Before cluster analysis, expression profiles of selected genes were scaled so that the mean and SD were equal to 0 and 1, respectively.

### TFBS enrichment analysis and pathway analysis

ENOCDE transcription factor ChIP-Seq datasets in the UCSC Genome Browser database and REFSEQ were used to extract the TFBS located up to 2 kbp upstream of transcription start site. Then, enrichment of TFBS in differentially expressed genes was analyzed by Fisher' exact test followed by multiple hypothesis testing correction by the FDR method. Condition-dependent TF usage was investigated by TFBS enrichment analysis. Enrichment score *s*, which is defined as *s* = 1 − FDR, was calculated and hierarchical clustering was performed.

### Network analysis and ARE prediction

Network and gene ontology annotation of genes was performed using the STRING, database version 9.1 (http://string-db.org/) [20]. The presence of ARE was predicted using the AREsite database (http://rna.tbi.univie.ac.at/cgi-bin/AREsite.cgi), which integrates a prediction based on the accessibility and evolutionary conservation of the ARE sites and experimentally validated target genes that are known to bind the ARE-binding proteins TTP, HuR and Auf1 [38].

### qRT-PCR

For cDNA synthesis, 500 ng of total RNA was reverse transcribed using the PrimeScript RT reagent Kit (Takara Corp., Otsu, Japan). cDNA equivalent to 5 ng of total RNA was used for all the PCR reactions. All the PCR reactions were performed using SYBR Premix Ex Taq (Takara Corp.) in a Thermal Cycler Dice Real Time System TP800 (TaKaRa). qRT-PCR was performed in duplicate for each sample using default two-step amplification procedures in accordance with the manufacturer's instructions. The standard curve method was used to determine the relative quantity of mRNA. All qRT-PCR data were normalized to GAPDH expression. PCR primers (PCReady primer) were purchased from Operon Biotechnology (Tokyo, Japan) and are described in Table S2.

### Determination of mRNA and phospho-protein duration and decay half-life rates

The half-life of mRNA or phospho-protein expression was calculated according to the methods used in previous studies [34,39].

We assumed a first-order decay of mRNA in the scheme:


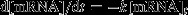
(1)


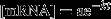
(2)

where the mRNA level [mRNA] is decreased with a decay constant *k*. Because we calculate the decay from the peak time point, *t_*peak, we therefore off-set:


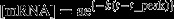
(3)

where *a* is the initial value of mRNA.

Measured mRNA data was fitted with Eqn (3) using the least squares method, and *k* and *a* were estimated using the generalized reduced gradient method in excel (Microsoft Corp, Redmond, WA, USA). mRNA half-life was calculated from the obtained *k* value using:


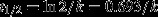
(4)

Calculated duration half-life values of more than 20 h were approximated as > 20 h for further analyses.

### Measurement of single cell ERK activity

Cells were seeded at a density of 8 × 10^3^ cells·well^−1^ of a 96-well plate. Prior to growth factor treatment, the cells were serum-starved for 16 h and then EGF was added. After a certain period, the cells were fixed with 3% paraformaldehyde/PBS for 30 min, permeabilized with 0.5% triton X-100/PBS for 5 min and blocked with 10% FBS/Blocking ONE solution (Nacalai Tesque, Kyoto, Japan) for 1 h. Next, the cells were immunostained using anti-phospho-ERK (Thr202/Tyr204) antibody (#4370; Cell Signaling Technology, Beverly, MA, USA) and cell nuclei were stained with 4′,6-diamidino-2-phenylindole (DAPI). Immunostained images and bright field images were photographed using In Cell Analyzer 2000 (GE Healthcare). Cell areas were automatically determined from bright field images, nuclear areas were determined from DAPI images and, finally, phospho-ERK intensities of each cell and nuclear region were calculated for at least 1200 cells in each condition. These image analyses were carried out using developer toolbox software (GE Healthcare).

### MCF-7 cell differentiation assay

Cells were seeded at a density of 3 × 10^4^ cells·well^−1^ of a 96-well plate and treated with growth hormone for 14 days with media changes every 2–3 days. For staining of accumulated lipid droplets, the cells were fixed with 4% neutralized formaldehyde in PBS and stained with an adipocyte fluorescence staining kit consisting of BODIPY (boron-dipyrromethene; a class of fluorescent dyes) and nuclear staining (Primary Cell Co., Ltd., Hokkaido, Japan) in accordance with the manufacturer's instructions. The cells were observed and photographed with a TCS-SPE microscope (Leica, Heidelberg, Germany). For quantitative analysis, the intensity of fluorescence was measured with a microflorescence reader; lipid droplets were detected at *D*_493_ and nuclear staining at *D*_461_. The ratio of *D*_493_/*D*_461_ was taken as the differentiation indicator for each cell line.

## Abbreviations

ActD: actinomycin D
ARE: AU-rich element
cDNA: complementary DNA
DAPI: 4′,6-diamidino-2-phenylindole
EGF: epidermal growth factor
EGFR: epidermal growth factor receptor
ERK: extracellular signal-regulated kinase
FDR: false discovery rate
HRG: heregulin
IEG: immediate early gene
JNK: c-Jun N-terminal kinase
MEK: mitogen-activated protein kinase kinase
NGF: nerve growth factor
qRT-PCR: quantitative real time-PCR
TFBS: transcription factor binding sites

## Supporting Information

**Fig. S1.** Time-course profiles of mRNA decay half-life of representative late response genes. **Table S1.** List of the ligand-responsive genes analyzed in the present study. **Table S2.** Sequences of PCR primers used in the present study.

## References

[1] Marshall CJ (1995). Specificity of receptor tyrosine kinase signaling: transient versus sustained extracellular signal-regulated kinase activation. Cell.

[2] Pouyssegur J, Lenormand P (2003). Fidelity and spatio-temporal control in MAP kinase (ERKs) signalling. Eur J Biochem.

[3] Ebisuya M, Kondoh K, Nishida E (2005). The duration, magnitude and compartmentalization of ERK MAP kinase activity: mechanisms for providing signaling specificity. J Cell Sci.

[4] Sasagawa S, Ozaki Y, Fujita K, Kuroda S (2005). Prediction and validation of the distinct dynamics of transient and sustained ERK activation. Nat Cell Biol.

[5] Santos SD, Verveer PJ, Bastiaens PI (2007). Growth factor-induced MAPK network topology shapes Erk response determining PC-12 cell fate. Nat Cell Biol.

[6] Nagashima T, Shimodaira H, Ide K, Nakakuki T, Tani Y, Takahashi K, Yumoto N, Hatakeyama M (2007). Quantitative transcriptional control of ErbB receptor signaling undergoes graded to biphasic response for cell differentiation. J Biol Chem.

[7] Nakakuki T, Birtwistle MR, Saeki Y, Yumoto N, Ide K, Nagashima T, Brusch L, Ogunnaike BA, Okada-Hatakeyama M, Kholodenko BN (2010). Ligand-specific c-Fos expression emerges from the spatiotemporal control of ErbB network dynamics. Cell.

[8] Murphy LO, Smith S, Chen RH, Fingar DC, Blenis J (2002). Molecular interpretation of ERK signal duration by immediate early gene products. Nat Cell Biol.

[9] Murphy LO, MacKeigan JP, Blenis J (2004). A network of immediate early gene products propagates subtle differences in mitogen-activated protein kinase signal amplitude and duration. Mol Cell Biol.

[10] Teramura Y, Ichinose J, Takagi H, Nishida K, Yanagida T, Sako Y (2006). Single-molecule analysis of epidermal growth factor binding on the surface of living cells. EMBO J.

[11] Hiroshima M, Saeki Y, Okada-Hatakeyama M, Sako Y (2012). Dynamically varying interactions between heregulin and ErbB proteins detected by single-molecule analysis in living cells. Proc Natl Acad Sci USA.

[12] Levkowitz G, Waterman H, Ettenberg SA, Katz M, Tsygankov AY, Alroy I, Lavi S, Iwai K, Reiss Y, Ciechanover A (1999). Ubiquitin ligase activity and tyrosine phosphorylation underlie suppression of growth factor signaling by c-Cbl/Sli-1. Mol Cell.

[13] Huang F, Kirkpatrick D, Jiang X, Gygi S, Sorkin A (2006). Differential regulation of EGF receptor internalization and degradation by multiubiquitination within the kinase domain. Mol Cell.

[14] Ross J (1995). mRNA stability in mammalian cells. Microbiol Rev.

[15] Gupta I, Clauder-Münster S, Klaus B, Järvelin AI, Aiyar RS, Benes V, Wilkening S, Huber W, Pelechano V, Steinmetz LM (2014). Alternative polyadenylation diversifies post-transcriptional regulation by selective RNA-protein interactions. Mol Syst Biol.

[16] Whelan JT, Hollis SE, Cha DS, Asch AS, Lee MH (2012). Post-transcriptional regulation of the Ras-ERK/MAPK signaling pathway. J Cell Physiol.

[17] Oksvold MP, Thien CB, Widerberg J, Chantry A, Huitfeldt HS, Langdon WY (2003). Serine mutations that abrogate ligand-induced ubiquitination and internalization of the EGF receptor do not affect c-Cbl association with the receptor. Oncogene.

[18] Ravid T, Heidinger JM, Gee P, Khan EM, Goldkorn T (2004). c-Cbl-mediated ubiquitinylation is required for epidermal growth factor receptor exit from the early endosomes. J Biol Chem.

[19] Goh LK, Huang F, Kim W, Gygi S, Sorkin A (2010). Multiple mechanisms collectively regulate clathrin-mediated endocytosis of the epidermal growth factor receptor. J Cell Biol.

[20] Franceschini A, Szklarczyk D, Frankild S, Kuhn M, Simonovic M, Roth A, Lin J, Minguez P, Bork P, von Mering C (2013). STRING v9.1: protein-protein interaction networks, with increased coverage and integration. Nucleic Acids Res.

[21] Kabnick KS, Housman DE (1988). Determinants that contribute to cytoplasmic stability of human c-fos and beta-globin mRNAs are located at several sites in each mRNA. Mol Cell Biol.

[22] Sawicki SG, Godman GC (1971). On the differential cytotoxicity of actinomycin D. J Cell Biol.

[23] Kuwano Y, Kim HH, Abdelmohsen K, Pullmann R, Martindale JL, Yang X, Gorospe M (2008). MKP-1 mRNA stabilization and translational control by RNA-binding proteins HuR and NF90. Mol Cell Biol.

[24] Bermudez O, Jouandin P, Rottier J, Bourcier C, Pagès G, Gimond C (2011). Post-transcriptional regulation of the DUSP6/MKP-3 phosphatase by MEK/ERK signaling and hypoxia. J Cell Physiol.

[25] Essafi-Benkhadir K, Pouysségur J, Pagès G (2010). Implication of the ERK pathway on the post-transcriptional regulation of VEGF mRNA stability. Methods Mol Biol.

[26] Zhai B, Yang H, Mancini A, He Q, Antoniou J, Di Battista JA (2010). Leukotriene B(4) BLT receptor signaling regulates the level and stability of cyclooxygenase-2 (COX-2) mRNA through restricted activation of Ras/Raf/ERK/p42 AUF1 pathway. J Biol Chem.

[27] Suer S, Ampasala D, Walsh MF, Basson MD (2009). Role of ERK/mTOR signaling in TGFbeta-modulated focal adhesion kinase mRNA stability and protein synthesis in cultured rat IEC-6 intestinal epithelial cells. Cell Tissue Res.

[28] Yang X, Wang W, Fan J, Lal A, Yang D, Cheng H, Gorospe M (2004). Prostaglandin A2-mediated stabilization of p21 mRNA through an ERK-dependent pathway requiring the RNA-binding protein HuR. J Biol Chem.

[29] Greenberg ME, Greene LA, Ziff EB (1985). Nerve growth factor and epidermal growth factor induce rapid transient changes in proto-oncogene transcription in PC12 cells. J Biol Chem.

[30] Sun H, Charles CH, Lau LF, Tonks NK (1993). MKP-1 (3CH134), an immediate early gene product, is a dual specificity phosphatase that dephosphorylates MAP kinase in vivo. Cell.

[31] Caunt CJ, Armstrong SP, Rivers CA, Norman MR, McArdle CA (2008). Spatiotemporal regulation of ERK2 by dual specificity phosphatases. J Biol Chem.

[32] Hao S, Baltimore D (2009). The stability of mRNA influences the temporal order of the induction of genes encoding inflammatory molecules. Nat Immunol.

[33] Sharova LV, Sharov AA, Nedorezov T, Piao Y, Shaik N, Ko MS (2009). Database for mRNA half-life of 19,977 genes obtained by DNA microarray analysis of pluripotent and differentiating mouse embryonic stem cells. DNA Res.

[34] Friedel CC, Dölken L, Ruzsics Z, Koszinowski UH, Zimmer R (2009). Conserved principles of mammalian transcriptional regulation revealed by RNA half-life. Nucleic Acids Res.

[35] Lindsten T, June CH, Ledbetter JA, Stella G, Thompson CB (1989). Regulation of lymphokine messenger RNA stability by a surface-mediated T cell activation pathway. Science.

[36] Chen CY, Gherzi R, Andersen JS, Gaietta G, Jürchott K, Royer HD, Mann M, Karin M (2000). Nucleolin and YB-1 are required for JNK-mediated interleukin-2 mRNA stabilization during T-cell activation. Genes Dev.

[37] Kim JH, Saito K, Yokoyama S (2002). Chimeric receptor analyses of the interactions of the ectodomains of ErbB-1 with epidermal growth factor and of those of ErbB-4 with neuregulin. Eur J Biochem.

[38] Gruber AR, Fallmann J, Kratochvill F, Kovarik P, Hofacker IL (2011). AREsite: a database for the comprehensive investigation of AU-rich elements. Nucleic Acids Res.

[39] Schwanhäusser B, Busse D, Li N, Dittmar G, Schuchhardt J, Wolf J, Chen W, Selbach M (2011). Global quantification of mammalian gene expression control. Nature.

