# Supplementary material for: Feedforward regulation of mRNA stability by prolonged extracellular signal-regulated kinase activity
Source: FEBS J. 2015 Jan 8;282(4):613–29. doi: 10.1111/febs.13172 (PMC4334673; doi:10.1111/febs.13172)
Supplement: Supplementary file 1 — Fig. S1. Time-course profiles of mRNA decay half-life of representative late response genes. Table S1. List of the ligand-responsive genes analyzed in the present study. Table S2. Sequences of PCR primers used in the present study. [file febs0282-0613-sd1.zip › febs13172-sup-0001-FigureS1-TabS1-S2.pdf]

the FEBS Journal
WILEY Blackwell
www.febsjournal.org

Feedforward regulation of mRNA stability by prolonged extracellular signal-regulated kinase activity

Takeshi Nagashima, Norihiko Inoue, Noriko Yumoto, Yuko Saeki, Shigeyuki Magi, Natalia Volinsky, Alexander Sorkin, Boris N. Kholodenko and Mariko Okada-Hatakeyama

DOI: 10.1111/febs.13172

Supplementary material

Feedforward regulation of mRNA stability by prolonged ERK activity

Takeshi Nagashima, Norihiko Inoue, Noriko Yumoto, Yuko Saeki, Shigeyuki Magi, Natalia Volinsky, Alexander Sorkin, Boris N. Kholodenko and Mariko Okada-Hatakeyama

Figure S1

Time-course profiles of mRNA decay half-life of representative late response genes.

The cells were stimulated with growth factor ligands (EGF or HRG) for 4 hours, then actinomycin D (ActD) (mRNA synthesis inhibitor) or U0126 (MEK inhibitor) was added. qRT-PCR was performed to obtain mRNA decay rate (decay half-life) as described in the Methods section.

Table S1

List of the ligand-responsive genes analyzed in the study.

Genes were selected as described in the Methods section. According to the peak timing, genes are classified into the early (peaked within 1. 5 hr), mid (peaked within 2-4 hrs) and late (peaked after 4 hrs) response group.

Table S2

Sequences of PCR primers used in the study.

Figure S1

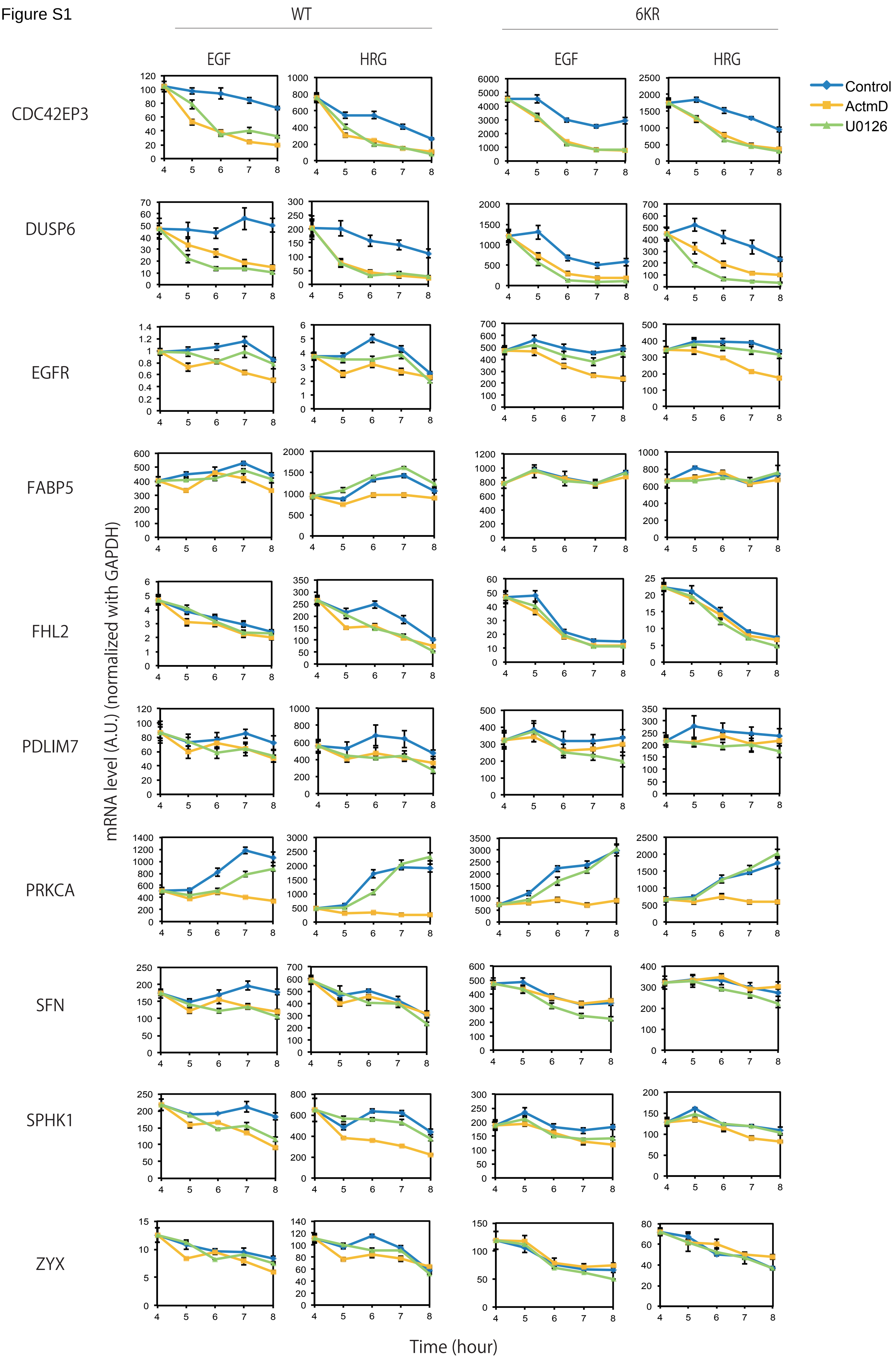
WT
6KR
EGF
HRG
EGF
HRG
Control
ActmD
U0126
CDC42EP3
DUSP6
EGFR
FABP5
FHL2
PDLIM7
PRKCA
SFN
SPHK1
ZYX
mRNA level (A.U.) (normalized with GAPDH)
Time (hour)

Table S1

Up-regulated genes and the peak time

Table S1

ProbeSetID	Symbol	Name	GeneID	Peak time in the cell-ligand conditions				Numbers of AU-rich sequences (W: A or T/U)								mRNA duration half-life (hour)			
				6KR EGF	6KR HRG	WT EGF	WT HRG	ATTTA	TTATTTATT	WTATTTATW	WWATTTAWW	WWTATTTATWW	WWWATTTAWWW	WWWTATTTATWWW	WWWWATTTAWWWW	6KR EGF	6KR HRG	MCF7 EGF	MCF7 HRG
218795_at	ACP6	acid phosphatase 6, lysophosphatidic	51205				late	1	0	0	0	0	0	0	0	17.22	81.91	256.83	699.23
202274_at	ACTG2	actin, gamma 2, smooth muscle, enteric	72		late			1	0	0	0	0	0	0	0	54.88	18.57	43.30	10.26
205891_at	ADORA2B	adenosine A2b receptor	136				late	1	0	0	0	0	0	0	0	4.74	2.39	11.18	3.94
219496_at	ANKRD57	ankyrin repeat domain 57	65124				early	0	0	0	0	0	0	0	0	6.36	3.11	10.57	2.17
39249_at	AQP3	aquaporin 3 (Gill blood group)	360	late				0	0	0	0	0	0	0	0	584.56	8.58	76.60	317.47
213618_at	ARAP2	ArfGAP with RhoGAP domain, ankyrin repeat and PH domain 2	116984		late			10	0	3	3	1	1	1	1	19.01	9.68	20.30	7.44
210090_at	ARC	activity-regulated cytoskeleton-associated protein	23237	early	early		mid	0	0	0	0	0	0	0	0	1.16	0.65	7.32	1.21
205239_at	AREG	amphiregulin	374			mid	mid	4	0	0	3	0	2	0	2	49.45	25.02	10.58	7.01
202672_s_at	ATF3	activating transcription factor 3	467	early	early		early	3	0	0	0	0	0	0	0	1.26	1.07	8.80	1.00
201170_s_at	BHLHE40	basic helix-loop-helix family, member e40	8553	early	early	early	early	0	0	0	0	0	0	0	0	5.48	2.66	4.52	2.74
212563_at	BOP1	block of proliferation 1	23246				late	0	0	0	0	0	0	0	0	182.00	241.38	8.13	772.77
219806_s_at	C11orf75	chromosome 11 open reading frame 75	56935				late	0	0	0	0	0	0	0	0	8.95	2.85	160.90	6.25
214696_at	C17orf91	chromosome 17 open reading frame 91	84981	early	early			0	0	0	0	0	0	0	0	8.20	4.33	5.17	3.91
217523_at	CD44	CD44 molecule (Indian blood group)	960	mid	mid			7	0	1	1	0	1	0	0	4.95	3.33	4.08	3.35
209286_at	CDC42EP3	CDC42 effector protein (Rho GTPase binding) 3	10602	late	mid		mid	6	0	0	1	0	1	0	0	14.18	6.26	7.38	4.53
209288_s_at	CDC42EP3	CDC42 effector protein (Rho GTPase binding) 3	10602	late	late	late	mid	6	0	0	1	0	1	0	0	22.62	9.12	5.00	3.15
219270_at	CHAC1	ChaC, cation transport regulator homolog 1 (E. coli)	79094		late		late	0	0	0	0	0	0	0	0	76.49	1347.31	47.42	971.86
209083_at	CORO1A	coronin, actin binding protein, 1A	11151				late	0	0	0	0	0	0	0	0	252.39	291.29	27.84	962.13
209101_at	CTGF	connective tissue growth factor	1490	early	early	early	early	3	0	0	3	0	1	0	1	0.69	0.42	0.76	0.88
217028_at	CXCR4	chemokine (C-X-C motif) receptor 4	7852				early	1	0	1	1	0	0	0	0	7.17	5.19	5.90	1.44
201289_at	CYR61	cysteine-rich, angiogenic inducer, 61	3491	early	early	early	early	5	0	0	0	0	0	0	0	0.93	0.84	1.51	1.32
210764_s_at	CYR61	cysteine-rich, angiogenic inducer, 61	3491	early	early		early	5	0	0	0	0	0	0	0	1.77	0.66	2.48	1.97
207147_at	DLX2	distal-less homeobox 2	1746		early	early		3	1	1	2	0	1	0	0	5.62	1.80	3.75	2.29
201041_s_at	DUSP1	dual specificity phosphatase 1	1843	early	early		early	4	2	2	2	0	0	0	0	1.52	0.97	12.09	0.78
204794_at	DUSP2	dual specificity phosphatase 2	1844	early	early	early	early	3	0	1	2	0	1	0	1	2.88	1.08	1.77	1.52
204014_at	DUSP4	dual specificity phosphatase 4	1846				early	6	1	1	1	0	0	0	0	17.17	13.66	9.71	4.36
204015_s_at	DUSP4	dual specificity phosphatase 4	1846				mid	6	1	1	1	0	0	0	0	9.81	6.60	9.14	5.61
209457_at	DUSP5	dual specificity phosphatase 5	1847	mid	early	early	mid	0	0	0	0	0	0	0	0	16.83	3.86	18.56	2.27
208891_at	DUSP6	dual specificity phosphatase 6	1848	late	late			1	0	0	0	0	0	0	0	72.30	603.24	165.69	6.77
208892_s_at	DUSP6	dual specificity phosphatase 6	1848	late	late			1	0	0	0	0	0	0	0	60.97	33.92	171.08	5.91
218995_s_at	EDN1	endothelin 1	1906	early	early		early	4	0	1	1	0	0	0	0	2.62	1.31	14.19	0.87
201983_s_at	EGFR	epidermal growth factor receptor	1956			late	late	9	0	0	0	0	0	0	0	96.97	71.55	3.67	3.13
201984_s_at	EGFR	epidermal growth factor receptor	1956	late	late			9	0	0	0	0	0	0	0	608.97	4.73	11.02	6.12
219232_s_at	EGLN3	egl nine homolog 3 (C. elegans)	112399		mid			4	0	0	2	0	1	0	0	7.27	4.25	13.65	6.94
201693_s_at	EGR1	early growth response 1	1958	early	early	early	early	1	0	0	1	0	1	0	0	1.48	0.67	4.69	0.63
201694_s_at	EGR1	early growth response 1	1958	early	early	early	early	1	0	0	1	0	1	0	0	6.44	2.15	16.76	1.59
205249_at	EGR2	early growth response 2	1959	early	early	early	early	2	0	0	0	0	0	0	0	0.73	0.64	0.60	0.46
206115_at	EGR3	early growth response 3	1960	early	early	early	early	3	0	0	0	0	0	0	0	1.73	1.34	1.08	1.67
207767_s_at	EGR4	early growth response 4	1961	early	early		early	0	0	0	0	0	0	0	0	0.94	0.63	5.35	1.04
207768_at	EGR4	early growth response 4	1961	early	early	early	early	0	0	0	0	0	0	0	0	0.73	0.60	0.90	1.11
214805_at	EIF4A1	Eukaryotic translation initiation factor 4A1	1973				early	1	0	0	0	0	0	0	0	4.12	5.17	6.03	0.73
214446_at	ELL2	elongation factor, RNA polymerase II, 2	22936	late				13	2	3	6	1	3	1	2	8.49	3.05	10.63	5.88
203499_at	EPHA2	EPH receptor A2	1969	mid	early		mid	3	1	2	3	1	2	0	0	8.21	2.93	11.69	1.75
201328_at	ETS2	v-ets erythroblastosis virus E26 oncogene homolog 2 (avian)	2114	mid	early			7	0	0	1	0	0	0	0	4.16	2.87	7.92	4.15
201329_s_at	ETS2	v-ets erythroblastosis virus E26 oncogene homolog 2 (avian)	2114	mid	early			7	0	0	1	0	0	0	0	4.24	1.83	7.76	2.66
206429_at	F2RL1	coagulation factor II (thrombin) receptor-like 1	2150	mid	mid		mid	3	0	0	0	0	0	0	0	3.37	1.60	3.39	1.62
213506_at	F2RL1	coagulation factor II (thrombin) receptor-like 1	2150	mid	mid	late	mid	3	0	0	0	0	0	0	0	4.21	2.16	3.03	2.19
202345_s_at	FABP5	fatty acid binding protein 5 (psoriasis-associated)	2171		late		late	0	0	0	0	0	0	0	0	417.65	12.70	238.62	3475.67
209210_s_at	FERMT2	fermitin family member 2	10979		mid			9	0	0	1	0	1	0	1	5.49	3.32	10.54	3.49
202949_s_at	FHL2	four and a half LIM domains 2	2274	mid	mid		late	6	0	1	3	1	2	1	1	7.02	3.03	17.74	2.27
209189_at	FOS	FBJ murine osteosarcoma viral oncogene homolog	2353	early	early	early	early	5	1	2	3	1	2	1	2	0.81	0.68	0.87	0.52
202768_at	FOSB	FBJ murine osteosarcoma viral oncogene homolog B	2354	early	early		early	3	0	0	1	0	0	0	0	0.92	0.78	4.80	0.62
204420_at	FOSL1	FOS-like antigen 1	8061	mid	early		mid	1	0	0	0	0	0	0	0	3.46	1.62	5.90	1.71
214240_at	GAL	galanin prepropeptide	51083	late	late		late	2	0	0	0	0	0	0	0	74.35	6.18	11.55	5684.75
204472_at	GEM	GTP binding protein overexpressed in skeletal muscle	2669	mid	early	early	mid	5	0	0	0	0	0	0	0	2.37	0.90	1.27	1.01
219936_s_at	GPR87	G protein-coupled receptor 87	53836	mid	mid		mid	0	0	0	0	0	0	0	0	6.88	3.36	5.48	1.99
203821_at	HBEGF	heparin-binding EGF-like growth factor	1839	mid	early		mid	5	0	2	4	1	2	1	1	2.37	1.88	4.28	1.17
38037_at	HBEGF	heparin-binding EGF-like growth factor	1839				mid	5	0	2	4	1	2	1	1	3.20	3.31	8.00	1.81
203394_s_at	HES1	hairy and enhancer of split 1, (Drosophila)	3280	early				0	0	0	0	0	0	0	0	1.80	1.84	14.33	3.28
214434_at	HSPA12A	heat shock 70kDa protein 12A	259217				late	5	0	0	0	0	0	0	0	20.14	9.45	33.57	6.03
208937_s_at	ID1	inhibitor of DNA binding 1, dominant negative helix-loop-helix pr	3397	early	early	early	early	1	0	0	0	0	0	0	0	6.63	2.62	34.24	27.18
202081_at	IER2	immediate early response 2	9592		early		early	0	0	0	0	0	0	0	0	7.22	3.46	7.74	2.99
201631_s_at	IER3	immediate early response 3	8870				mid	6	0	2	3	2	3	1	1	17.01	10.35	13.60	3.88
222062_at	IL27RA	interleukin 27 receptor, alpha	9466				late	0	0	0	0	0	0	0	0	271.93	6.28	515.58	11.12
205945_at	IL6R	interleukin 6 receptor	3570				mid	6	0	0	0	0	0	0	0	6.70	2.30	5.61	2.23
210511_s_at	INHBA	inhibin, beta A	3624	late			late	13	2	4	6	1	3	1	1	3.18	4.91	34.08	3.30
204698_at	ISG20	interferon stimulated exonuclease gene 20kDa	3669				late	3	0	0	0	0	0	0	0	16.33	29.10	178.16	1431.31
33304_at	ISG20	interferon stimulated exonuclease gene 20kDa	3669				late	3	0	0	0	0	0	0	0	1600.71	39.20	NA	1495.80
201656_at	ITGA6	integrin, alpha 6	3655	late	late		late	8	0	0	1	0	0	0	0	837.54	42.46	26.78	9.27
215177_s_at	ITGA6	integrin, alpha 6	3655	late	late			8	0	0	1	0	0	0	0	1291.61	5.69	8.84	5.82
208083_s_at	ITGB6	integrin, beta 6	3694	late				9	0	0	2	0	0	0	0	5.00	22.36	4.11	3.32
201464_x_at	JUN	jun proto-oncogene	3725		early			3	0	1	1	1	1	0	0	9.90	2.89	28.58	3.64
201465_s_at	JUN	jun proto-oncogene	3725		early			3	0	1	1	1	1	0	0	5.82	2.83	17.00	4.63
201466_s_at	JUN	jun proto-oncogene	3725	early	early			3	0	1	1	1	1	0	0	3.82	1.84	9.38	1.13
201473_at	JUNB	jun B proto-oncogene	3726		early		early	2	0	0	0	0	0	0	0	5.09	2.06	8.46	2.13
213146_at	KDM6B	lysine (K)-specific demethylase 6B	23135				mid	3	0	0	2	0	2	0	0	3.00	1.74	7.68	1.57
41386_i_at	KDM6B	lysine (K)-specific demethylase 6B	23135				mid	3	0	0	2	0	2	0	0	4.11	2.08	17.17	1.80
212942_s_at	KIAA1199	KIAA1199	57214	late				4	1	1	1	1	1	1	1	580.52	9.12	58.84	5.75
202393_s_at	KLF10	Kruppel-like factor 10	7071	early	early		early	3	0	0	2	0	1	0	0	2.61	1.32	3.21	2.46
208960_s_at	KLF6	Kruppel-like factor 6	1316	early	early		early	6	0	0	0	0	0	0	0	8.97	1.46	11.26	1.76
208961_s_at	KLF6	Kruppel-like factor 6	1316				early	6	0	0	0	0	0	0	0	6.43	1.18	18.15	1.44
211610_at	KLF6	Kruppel-like factor 6	1316				early	6	0	0	0	0	0	0	0	19.38	13.03	14.48	2.53
209125_at	KRT6A	keratin 6A	3853				late	1	0	0	0	0	0	0	0	36.77	64.42	117.04	931.72

213711_at	KRT81	keratin 81	3887				late	0	0	0	0	0	0	0	0	258.24	144.34	211.32	816.89
215189_at	KRT86 /// LOC100	keratin 86 /// hypothetical LOC100509764	100509764 //	late	late		late	0	0	0	0	0	0	0	0	683.70	70.90	151.70	1503.20
202267_at	LAMC2	laminin, gamma 2	3918				late	4	0	1	2	0	0	0	0	174.39	13.51	60.59	6.91
207517_at	LAMC2	laminin, gamma 2	3918				mid	4	0	1	2	0	0	0	0	9.41	3.48	13.62	3.08
205266_at	LIF	leukemia inhibitory factor (cholinergic differentiation factor)	3976	mid	mid		mid	7	2	3	5	1	1	1	1	4.34	4.46	8.75	2.45
203570_at	LOXL1	lysyl oxidase-like 1	4016	late	late		late	0	0	0	0	0	0	0	0	801.33	418.44	314.42	1276.89
202998_s_at	LOXL2	lysyl oxidase-like 2	4017	late				3	0	0	1	0	0	0	0	1178.43	190.97	63.01	156.61
213909_at	LRRC15	leucine rich repeat containing 15	131578				late	4	0	0	0	0	0	0	0	151.94	55.12	8.34	23.26
215375_x_at	LRRFIP1	Leucine rich repeat (in FLII) interacting protein 1	9208				early	6	0	0	2	0	0	0	0	4.90	3.29	7.37	0.22
205193_at	MAFF	v-maf musculoaponeurotic fibrosarcoma oncogene homolog F (av	23764				mid	4	2	2	2	1	1	0	0	4.56	2.48	6.01	1.57
36711_at	MAFF	v-maf musculoaponeurotic fibrosarcoma oncogene homolog F (av	23764	mid	early	early	mid	4	2	2	2	1	1	0	0	2.16	1.31	1.52	1.18
209373_at	MALL	mal, T-cell differentiation protein-like	7851	late	late		late	3	0	0	0	0	0	0	0	5780.43	14.84	10.91	75.32
215498_s_at	MAP2K3	mitogen-activated protein kinase kinase 3	5606		mid		mid	1	0	0	0	0	0	0	0	8.17	6.29	356.18	3.90
205192_at	MAP3K14	mitogen-activated protein kinase kinase kinase 14	9020				mid	0	0	0	0	0	0	0	0	4.23	2.41	16.89	1.24
221467_at	MC4R	melanocortin 4 receptor	4160				late	0	0	0	0	0	0	0	0	60.53	21.04	4.68	2.98
200796_s_at	MCL1	myeloid cell leukemia sequence 1 (BCL2-related)	4170	mid	early			10	0	2	3	0	0	0	0	3.59	1.71	2.99	2.07
200798_x_at	MCL1	myeloid cell leukemia sequence 1 (BCL2-related)	4170	mid	early			10	0	2	3	0	0	0	0	4.83	2.76	5.06	2.99
204475_at	MMP1	matrix metallopeptidase 1 (interstitial collagenase)	4312	late			late	3	0	1	1	0	0	0	0	5569.52	6.49	270.14	27250.35
203780_at	MPZL2	myelin protein zero-like 2	10205	late	late			6	0	1	3	1	1	0	0	733.70	2.59	55.12	5.04
214385_s_at	MUC5AC	mucin 5AC, oligomeric mucus/gel-forming	4586				early	0	0	0	0	0	0	0	0	283.29	129.13	NA	1021.80
202431_s_at	MYC	v-myc myelocytomatosis viral oncogene homolog (avian)	4609		early			4	0	0	0	0	0	0	0	3.17	0.96	5.99	2.09
202340_x_at	NR4A1	nuclear receptor subfamily 4, group A, member 1	3164	early	early	early	early	3	0	0	1	0	1	0	1	0.82	0.87	0.97	0.91
211143_x_at	NR4A1	nuclear receptor subfamily 4, group A, member 1	3164	early	early			3	0	0	1	0	1	0	1	3.98	1.29	8.09	5.46
204621_s_at	NR4A2	nuclear receptor subfamily 4, group A, member 2	4929	early	early		early	4	0	0	1	0	1	0	0	1.29	0.73	8.13	0.77
204622_x_at	NR4A2	nuclear receptor subfamily 4, group A, member 2	4929	early	early		early	4	0	0	1	0	1	0	0	1.01	1.44	3.39	0.48
216248_s_at	NR4A2	nuclear receptor subfamily 4, group A, member 2	4929	early	early		early	4	0	0	1	0	1	0	0	1.06	1.48	3.66	0.40
207978_s_at	NR4A3	nuclear receptor subfamily 4, group A, member 3	8013	early	early		early	0	0	0	0	0	0	0	0	0.85	0.59	3.82	0.99
209959_at	NR4A3	nuclear receptor subfamily 4, group A, member 3	8013	early	early		early	0	0	0	0	0	0	0	0	0.58	0.84	1.28	1.01
206825_at	OXTR	oxytocin receptor	5021	late	late			5	0	0	1	0	0	0	0	14.05	9.62	43.30	3.96
213684_s_at	PDLIM5	PDZ and LIM domain 5	10611		early		early	19	0	2	4	2	3	0	1	3.68	2.40	7.45	1.69
203370_s_at	PDLIM7	PDZ and LIM domain 7 (enigma)	9260				late	0	0	0	0	0	0	0	0	296.13	8.20	246.83	70.46
217996_at	PHLDA1	pleckstrin homology-like domain, family A, member 1	22822	mid	mid	mid	mid	14	1	1	2	1	1	0	0	5.08	3.00	2.07	1.61
217997_at	PHLDA1	pleckstrin homology-like domain, family A, member 1	22822	mid	mid	mid	mid	14	1	1	2	1	1	0	0	2.75	2.05	1.77	1.56
217999_s_at	PHLDA1	pleckstrin homology-like domain, family A, member 1	22822	mid	mid		mid	14	1	1	2	1	1	0	0	6.90	3.12	5.05	1.82
218000_s_at	PHLDA1	pleckstrin homology-like domain, family A, member 1	22822	mid	mid		mid	14	1	1	2	1	1	0	0	4.44	3.14	5.84	2.02
201860_s_at	PLAT	plasminogen activator, tissue	5327				early	5	0	2	2	0	0	0	0	65.39	6.92	19.86	3.43
218223_s_at	PLEKHO1	pleckstrin homology domain containing, family O member 1	51177		mid			2	0	0	0	0	0	0	0	8.65	4.99	17.85	4.74
204285_s_at	PMAIP1	phorbol-12-myristate-13-acetate-induced protein 1	5366				early	5	1	1	1	1	1	1	1	6.44	6.13	5.40	2.65
213093_at	PRKCA	protein kinase C, alpha	5578		late			7	5	5	6	4	5	4	4	347.26	5.46	189.30	286.12
202388_at	RGS2	regulator of G-protein signaling 2, 24kDa	5997	mid				2	0	0	1	0	1	0	1	4.21	4.04	12.51	2.32
204268_at	S100A2	S100 calcium binding protein A2	6273	late				0	0	0	0	0	0	0	0	539.51	8.40	73.38	511.22
209260_at	SFN	stratifin	2810				late	0	0	0	0	0	0	0	0	37.95	14.44	101.92	8.05
208078_s_at	SIK1	salt-inducible kinase 1	150094	early	early		early	3	0	0	2	0	2	0	2	0.92	0.85	3.49	0.59
202236_s_at	SLC16A1	solute carrier family 16, member 1 (monocarboxylic acid transpo	6566	late	late			6	0	0	1	0	1	0	1	647.64	6.01	11.54	11.80
209921_at	SLC7A11	solute carrier family 7, (cationic amino acid transporter, y+ syster	23657		late			6	0	0	1	0	0	0	0	8.28	602.49	NA	787.26
201195_s_at	SLC7A5	solute carrier family 7 (cationic amino acid transporter, y+ syster	8140		late			2	1	1	2	1	1	0	0	101.45	457.74	11.23	610.12
222071_s_at	SLCO4C1	solute carrier organic anion transporter family, member 4C1	353189		late			20	0	0	4	0	2	0	1	7.28	3.42	6.65	11.24
202935_s_at	SOX9	SRY (sex determining region Y)-box 9	6662			late	late	8	1	1	2	1	2	1	2	4.81	6.53	4.60	4.84
202936_s_at	SOX9	SRY (sex determining region Y)-box 9	6662		mid		late	8	1	1	2	1	2	1	2	15.73	7.69	11.99	3.54
219257_s_at	SPHK1	sphingosine kinase 1	8877				late	0	0	0	0	0	0	0	0	9.86	7.02	12.08	14.40
205064_at	SPRR1B	small proline-rich protein 1B	6699				late	0	0	0	0	0	0	0	0	155.93	101.38	178.83	102.63
202401_s_at	SRF	serum response factor (c-fos serum response element-binding tra	6722		early			3	1	1	1	0	0	0	0	4.59	4.57	16.26	3.99
201516_at	SRM	spermidine synthase	6723				late	1	0	0	0	0	0	0	0	127.62	55.61	219.89	715.45
202786_at	STK39	serine threonine kinase 39	27347		late			9	1	2	3	0	0	0	0	134.19	4.21	32.21	137.29
203085_s_at	TGFB1	transforming growth factor, beta 1	7040				late	1	0	0	0	0	0	0	0	121.61	37.50	339.34	NA
209386_at	TM4SF1	transmembrane 4 L six family member 1	4071	late	late		late	9	0	0	1	0	0	0	0	13.42	5.57	54.74	2.40
209387_s_at	TM4SF1	transmembrane 4 L six family member 1	4071	late	late			9	0	0	1	0	0	0	0	3761.46	2.50	14.92	7.33
215034_s_at	TM4SF1	transmembrane 4 L six family member 1	4071	late	late			9	0	0	1	0	0	0	0	3542.53	5.21	53.80	6.85
213338_at	TMEM158	transmembrane protein 158 (gene/pseudogene)	25907		late			0	0	0	0	0	0	0	0	471.42	5.65	8.86	6.94
220177_s_at	TMPRSS3	transmembrane protease, serine 3	64699			early	mid	0	0	0	0	0	0	0	0	4.76	4.70	62.83	8.41
214581_x_at	TNFRSF21	tumor necrosis factor receptor superfamily, member 21	27242	late	mid	late	late	6	0	0	1	0	0	0	0	21.06	3.86	2.17	1.93
218856_at	TNFRSF21	tumor necrosis factor receptor superfamily, member 21	27242	late	mid		late	6	0	0	1	0	0	0	0	9.65	5.94	5.11	3.35
211828_s_at	TNIK	TRAF2 and NCK interacting kinase	23043	late				7	0	0	2	0	0	0	0	5.62	20.31	5.32	8.21
213107_at	TNIK	TRAF2 and NCK interacting kinase	23043	late	late		late	7	0	0	2	0	0	0	0	1194.12	6.23	22.67	8.48
202241_at	TRIB1	tribbles homolog 1 (Drosophila)	10221	early	early		early	1	0	0	0	0	0	0	0	3.86	1.32	5.20	1.84
204141_at	TUBB2A	tubulin, beta 2A	7280				late	0	0	0	0	0	0	0	0	196.33	8.54	172.44	26.81
200931_s_at	VCL	vinculin	7414		early			2	0	0	0	0	0	0	0	7.87	5.44	5.80	4.92
201531_at	ZFP36	zinc finger protein 36, C3H type, homolog (mouse)	7538		early			5	1	1	1	0	0	0	0	8.35	2.86	13.11	3.13
206448_at	ZNF365	zinc finger protein 365	22891	mid	mid		late	0	0	0	0	0	0	1	0	5.67	4.42	5.10	4.50
200808_s_at	ZYX	zyxin	7791	late	mid		late	0	0	0	0	0	0	0	0	17.93	6.03	26.15	5.50
215706_x_at	ZYX	zyxin	7791	late	mid		late	0	0	0	0	0	0	0	0	16.17	5.03	8.39	5.26
215029_at	---	---	---				early	0	0	0	0	0	0	0	0	8.00	16.12	4.56	0.43
215401_at	---	---	---				early	0	0	0	0	0	0	0	0	3.54	1.40	4.40	0.37
215635_at	---	---	---				early	0	0	0	0	0	0	0	0	9.28	59.49	8.46	0.95
216766_at	---	---	---				early	0	0	0	0	0	0	0	0	12.43	97.85	2.07	0.40
222303_at	---	---	---	early	early		early	0	0	0	0	0	0	0	0	1.80	0.47	6.70	0.73

Table S2

Sequences of PCR primers used in the study are as follows;

FHL2	Fw: TGG CAT AAC GAC TGC TTT AAC TGT A
	Rev: GTG TGA GAT CAC AAG CAG CAA
EGFR	Fw: GCG GGA CAT AGT CAG CAG TG
	Rev: TTT GGT CAG TTT CTG GCA GTT C
CDC42EP3	Fw: AAT GAG TTC TTC CGG GCC AA
	Rev: GAT CCT CCA ATG GTC GGG AG
DUSP6	Fw: CTG GAA GGT GGC TTC AGT AAG T
	Rev: CTC GAT GTC CGA GGA AGA GTC
PRKCA	Fw: CTT CTT CCG GAG GAT CGA CTG
	Rev: AGT TCT CTG CTC CTT TGC CAC
FABP5	Fw: GAA ACC ACA GCT GAT GGC AG
	Rv: TGT TCA TGA CAC ACT CCA CCA C
PDLIM7	Fw: GAT GGC GAG AAT GCG GGT AG
	Rv: GCC TTC TGC GGT TTG CTC TG
SFN	Fw: ACT ACG AGA TCG CCA ACA GC
	Rv: CAG TGT CAG GTT GTC TCG CA
SPHK1	Fw: GGT GTG TTT GCA GTG GAT GG
	Rv: AGT AGT TTG GGT GCA CCT GG
ZYX	Fw: ATC ACT GAC CGC ATG CTG AG
	Rv: GTA CTG CTT GTG GTA GTC GGG
GAPDH	Fw: GCA CCG TCA AGG CTG AGA AC
	Rev: ATG GTG GTG AAG ACG CCA GT
